# Growth performance, behavior, gene expression, carcass characteristics, stress indicators, and economical parameters of avian 48 broiler chickens raised under three different stocking density

**DOI:** 10.3389/fvets.2025.1517142

**Published:** 2025-04-10

**Authors:** Shimaa A. Sakr, Ahmed F. Abouelnaga, Ahmed I. Ateya, Nada M. A. Hashem, Noha M. Wahed, Ibrahim F. Rehan, Asmaa Elnagar, František Zigo, Illia Siedoi, Walied A. Kamel, Huda A. El-Emam

**Affiliations:** ^1^Department of Animal Wealth Development, Faculty of Veterinary Medicine, Mansoura University, Mansoura, Egypt; ^2^Department of Animal Husbandry, Faculty of Veterinary Medicine, Mansoura University, Mansoura, Egypt; ^3^Department of Physiology, Faculty of Veterinary Medicine, Mansoura University, Mansoura, Egypt; ^4^Department of Husbandry and Development of Animal Wealth, Faculty of Veterinary Medicine, Menoufia University, Menoufia, Egypt; ^5^Department of Pathobiochemistry, Faculty of Pharmacy, Meijo University Yagotoyama, Nagoya-shi, Japan; ^6^Department of Nutrition and Animal Husbandry, University of Veterinary Medicine and Pharmacy, Košice, Slovakia; ^7^Department of Zoology, Faculty of Science, Mansoura University, Mansoura, Egypt; ^8^Laboratory of Fundamental Oncology, National Cancer Center Research Institute, Chuo-ku, Japan

**Keywords:** stocking density, growth performance, behavior, genetics, stress, meat quality, broiler

## Abstract

The current research evaluated the consequence of varying stocking densities on growth performance, carcass features, hematological, welfare, economic parameters, and immune markers of broiler chicks. A total of 324 Avian 48 were haphazardly classified into three different stocking densities. There were 14 birds/m^2^ in the low stocking density (LSD) group, 18 birds/m^2^ in the medium stocking density (MSD) group, and 22 in the high stocking density (HSD) group. Compared to the other two groups, the HSD birds’ body weight and daily weight gain were significantly lower (*p* < 0.05). The LSD group demonstrated a significant increase in productive efficiency (EPEF and EBI) compared to the medium and high SD groups (*p* < 0.003). The birds from the HSD group exhibited the lowest values for carcass characteristics compared to the low and medium SD groups. At the hematological level, the HSD group exhibited significantly elevated levels of HB, RBCs, heterophils, and lymphocytes compared to the LSD and MSD groups (*p* < 0.011, *p* < 0.0001, and *p* < 0.0001), respectively. Compared to the LSD group, the levels of cortisol, a hallmark of oxidative stress, were considerably greater in the MSD and HSD groups (*p* < 0.0001). Concerning gene expression, the birds in the LSD group exhibited a significant improvement in growth, intestinal health, and anti-inflammatory genes compared to the MSD and HSD groups. In addition, inflammatory markers were significantly downregulated. The HSD group exhibited the lowest net profit compared to the other groups (*p* < 0.0001). At the behavioral level, birds in the LSD group demonstrated a significantly shorter TI duration (*p* < 0.0001) and latency (*p* < 0.043) in OFT to the first step, lower mobility duration (*p* < 0.004), and pecking (*p* < 0.05) compared to other groups. Our study concluded that rearing in LSD up to MSD could be applied without compromising broiler performance.

## 1 Introduction

There has been an increased demand for broiler meat in the past decade owing to its excellent quality, low-fat content, and high protein levels. Researchers are investigating methods to reduce production costs to maximize broiler meat yield within the lowest feasible floor area (1). Recently, the global poultry business’s primary objective has been to increase the productivity of broiler meat (kg) per m^2^ with superior quality and prevent losses in production caused by overloading. Globally, broiler stocking density in poultry commerce has significantly increased to improve productivity, optimize the utilization of limited space, reduce production costs (leading to increased income), and address health and welfare issues. On the other hand, environmental conditions, such as stocking density, heat stress, and nutritional and metabolic illnesses brought on by poor chicken management, can directly affect the phenotypic expression of genetically comparable birds (2). This results in genotype-by-environment interaction (G × E), which is characterized by genetically identical organisms exhibiting distinct phenotypic expressions of the same trait in various circumstances (3). To address concerns about animal welfare, traditional broiler farming must change to more animal-friendly production methods. Two of the most promising elements that will enable this transition are genetics and stocking density (4). Further evidence emphasized that restricted space for birds is believed to be a significant factor that affects their wellbeing, physical activity, and broiler production systems (5, 6). There is evidence that limited space for birds not only influences growth performance but also modifies the behavior, endocrinology, carcass, and meat quality features (7), which can harm poultry health. These effects include increased ammonia production, respiratory problems, cannibalism, footpad dermatitis, and high litter moisture. Restricted space also affects the immune system, causing stress, and impacts welfare by limiting the ability of broilers to preen in the litter-flooring system. Additionally, it affects air quality by reducing air exchange at the bird level and hampering the dissipation of body heat in high environmental temperatures. Furthermore, restricted space has adverse effects on productive performance, including weight gain, feed intake, feed conversion ratio, carcass characteristics, and mortality rates (8–10). Stocking density varies across different world regions based on distinct criteria (11). Density/m^2^ varies from 11 to 20 chicks/m^2^ depending on parameters including breed type, availability of open area, and slaughter age (12). In warm areas, the most often utilized densities are 30–42 kg live weight/m^2^ and 30 kg live weight/m^2^ (13). The SD should also be optimized for the produced live body weight/m^2^ of the raising area of the poultry house or cage (14). Several authors have reported no impact of lowering stocking density on chicken performance (15). However, some studies have reported that decreasing stocking density might improve the performance of broilers (16). It is hypothesized that the balance of laying chickens’ physiological and immunological reactions is maintained through the hypothalamic–pituitary–adrenal pathways, which are triggered by the stress of high stocking density (17). Regarding the health and immunological function of laying hens, the heterophil/lymphocyte (H/L) ratio is thought to be a sign of ongoing stress (18). The primary purpose of blood biochemical profiles is to serve as an indicator of the physiological and metabolic state of broilers. In addition, broilers with high stocking density exhibited metabolic changes in blood biochemical parameters, including increased heterophiles. These changes are accompanied by an elevated heterophile-to-lymphocyte ratio, decreased lymphocytes (19), elevated blood stress hormones (20), alleviated immune response (21), increased oxidative stress (22), increased vulnerability to infection as in Newcastle disease and necrotic enteritis (23), and elevated cortisol, cholesterol, and glucose levels in the plasma during the adaptation phase of stress. In the broiler sector, carcass performance is a critical economic element (24). Therefore, the objective of the present study is to clarify the possible effects of various stocking densities on performance, carcass characteristics, immunity, behavioral and welfare assessments, oxidative stress, hematological, biochemical, and gene expression profiles of immune and growth markers, as well as economic parameters of Avian 48 broiler breeds, to determine the optimal stocking density that can enhance growth performance, improve meat quality, and optimize welfare while simultaneously minimizing production costs and negative consequences that arise from high stocking densities.

## 2 Materials and methods

### 2.1 Animal ethical approval statement

The recent study was authorized by the Medical Research Ethics Committee of Mansoura University, Egypt (R/119/2022). The study was conducted at the Research Unit, Faculty of Veterinary Medicine, Mansoura University, Egypt.

### 2.2 Experimental animal design

Three hundred and twenty-four one-day-old (45 g) chicks were randomly divided into three experimental groups, each including six replicates with varying stocking densities. The 1st group (LSD) consisted of 14 birds/m^2^ (28 kg/m^2^), the 2nd group (MSD) comprised 18 birds/m^2^ (36 kg/m^2^), and the 3rd group (HSD) included 22 birds/m^2^ (44 kg/m^2^). Each pen had a flexible side that enabled modifying the space allotted for birds. The chicks underwent daily cleanings and were provided with a deep litter floor of 10–15 cm wood shavings, unlimited access to a standard diet formulated according to (25), and freshwater. The photoperiod was 16 L:8D. The broilers in each group were held in a pen with similar conditions. Throughout the first week, the room temperature was maintained at 32°C, and by the end of the third week, it had progressively dropped to 24°C. The temperature remained constant at 24°C until the end of the study (42 d). The relative humidity varied between 67 and 77% throughout the trial. The chicks were raised under the same managerial conditions. As part of standard immunization procedures, all chickens received vaccinations against Newcastle, Gumboro, and infectious bronchitis. The feeding schedule was allotted into three feeding phases: from zero to the 14th day of age. The grower phase spanned from the 15th to the 28th days of age, whereas the finisher phase was from the 29th to the 41st days of age.

### 2.3 Growth performance

The weight of the chickens and feed from each group was measured weekly until the sixth week of their lifespan. Feed intake (FI), body weight (BW), body weight gain (BWG), and feed conversion ratio (FCR) were determined for each feeding period using the techniques described previously (26).

### 2.4 Carcass traits and meat quality

At the end of the experiment, three birds were chosen randomly from each replication and then slaughtered following 12 h of fasting. The hot carcass was weighed and recorded after exsanguination, plucking, and gutting. Edible parts (breast, thigh, liver, heart, and gizzard) were separately weighed and recorded. The dressed weight was estimated as % = carcass weight x 100/live body weight. Muscle samples of breasts and thighs (20 g of each) were stored in a deep freezer (−20°C) and then defrosted in a refrigerator (4°C) for 24 h to determine drip. The pH values of a 10-g muscle sample were determined after it was refrigerated for 24 h in a solution containing 1 g of homogenized muscle (breast/thigh) and 40 mL of deionized water. After pouring the mixed liquid into a transparent glass for 30 s, a Knick digital pH meter (standardized to pH 4.01, 7.00, and 10.01 standard buffers) was used to insert the pH meter electrode (27). Drip loss was the difference in weight between the frozen and thawed samples. Blotting dry with filter paper was used to quantify the drip loss (28). Furthermore, the weight differential between the raw fresh sample (20 g of each) and cooked samples is referred to as the cooking loss (29).

### 2.5 Behavioral and welfare assessments

#### 2.5.1 Behavior and fear reactions

To eliminate any potential effect of blood sampling on behavior, the scheduled time of behavioral observations was 2 days after the end of blood sampling. All behavioral observation tests were conducted under the same light color and intensity as in the experimental design. Behavioral observations were conducted on 10 randomly selected birds/replicates. The operator refrained from making any unnecessary noise or movement to prevent the bird from being distracted during testing.

#### 2.5.2 Tonic immobility test

The birds were positioned in an isolated room and exposed to the immobility test. The TI was encouraged by overturning our bird on its backside on a clean bench with its neck resting over a U-shaped cloth and manually performing a 10-s restraint till our bird stopped struggling. The immobility duration was determined as the time from the bird becoming immovable till the bird regained an upright position. The observer avoided direct eye contact with the bird as it had been established to lengthen the immobility time. The measurement of TI was completed if the bird continued in the immobility status for 600 s (TI = 600) or if it failed to persist in the immobility status after five unsuccessful attempts (TI = 0). For every bird, the number of attempts needed to complete TI was noted (30).

### 2.6 Open field test

Every bird was tested in a 1 × 1 m arena, and throughout the 3-min testing period, behavioral observations were recorded. The area was equally divided into four sectors by a vertical and horizontal line. Each bird was always positioned in sector 1 of the area. The number of visited sectors, first step latency, duration of immobility (both duration of sitting and standing), duration of walking, floor or wall pecking, and elimination frequency (droppings) were videotaped (31).

### 2.7 Hematological, biochemical, and oxidative stress

Blood samples (about 3 mL up to 5 mL) from all groups (3/replicate) were assembled at the end of the experiment. A plastic syringe was utilized to collect blood samples, and a centrifuge was used to separate the blood. Blood samples were gathered from the wing vein in separate labeled test tubes. Two aseptic blood samples, one with and one without an anticoagulant, were carefully drawn from the wing vein. The samples with anticoagulants were used to determine hemoglobin (Hb), erythrocytes (RBCs), leucocytes (WBCs), and differential leucocytic counts. In contrast, the samples without anticoagulant were permitted to clot and were then centrifuged for 15 min at 4,000 rpm for serum extraction. Cortisol, malondialdehyde (MDA), total antioxidant capacity (TAC), immunoglobulin G (Ig G), total protein, globulin, and albumin, in addition to thyroxine hormone (T4), were determined.

### 2.8 Gene expression of immune and growth markers

Collected tissues (liver and spleen) from 3 randomly selected birds of each replicate (*n* = 18 birds) per treatment were used to extract total RNA using Trizol reagent (easy-RED™, iNtRON Biotechnology) according to the manufacturer’s instructions. The NanoDrop^®^ ND-1000 spectrophotometer was utilized to measure the quantity of extracted RNA. Using the QuantiTect Reverse Transcription kit (Qiagen, Heidelberg, Germany), the first strand of cDNA was synthesized from the attained RNA by following the manufacturing protocols. Guidelines for biosecurity and infection control outlined the protocols for all laboratory operations following the guidelines for the Veterinary Personal Biosecurity & Infection Control manual. Using SYBR.

Green PCR Master Mix (2x SensiFast™ SYBR, Bioline, catalog No. Bio-98002), relative quantification of the mRNA levels of growth (IGF1 and GH), intestinal (Muc2, Cath-B, *Calbindin*, and *Gastrotropin*), and inflammatory (IL-6, IL-8, IL-10, and IL-13) genes was carried out via real-time PCR. A total of 20 μL was used for the reaction mixture, which included 10 μL of 2x SensiFast SYBR, 3 μL of cDNA, 5.4 μL of H_2_O, and 0.8 μL of each primer. The real-time PCR experiments were conducted following the protocols outlined previously (32). The PCR cycling conditions were as follows: 45 cycles of 94°C for 15 s, annealing temperatures as listed in Table 1 for 20 s, and 72°C for 20 s. The cycle started at 95°C for 10 min. A melting curve analysis was carried out at the end of the amplification stage to verify the PCR product’s specificity. The 2-ΔΔCt technique was utilized to determine the relative expression of each gene in every sample following normalization to the housekeeping GAPDH gene (33).

**Table 1:** Primers’ sequence and melting temperature used in real-time quantitative PCR.

Genes	Isolation source	Primer sequence (5′–3′)	Annealing temperature (°C)	Accession number	References
*IGF-1*	Liver	F: ACCTTGGCCTGTGTTTGCTTAC	60	NM_001004384	(83)
		R: AGCCTCTGTCTCCACATACGAAC			
*GH*	Liver	F: CACCACAGCTAGAGACCCACATC	60	HE608816	(105)
		R: CCCACCGGCTCAAACTGC			
*IL-6*	Spleen	F: CCCTCACGGTCTTCTCCATA		NM_204628.1	(106)
		R: CTCCTCGCCAATCTGAAGTC	58		
*IL-10*	Spleen	F: GGAGCTGAGGGTGAAGTTTG	62	NM_001004414.2	(106)
		R: TAGAAGCGCAGCATCTCTGA			
*IL-8*	Spleen	F: GCTGATCGTAAAGGCACTTATG		NM_205498.1	(107)
		R: GTGAAAGGTGGAAGATGGAATG	56		
*IL-13*	Spleen	F: CTGCCCTTGCTCTCCTCTGT	60	AJ621250.1	(108)
		R: CCTGCACTCCTCTGTTGAGCTT			
*Cath-B1*	Ileum	F:CCGTGTCCATAGAGCAGCAG	62	NM_001271172.1	(109)
		AGTGCTGGTGACGTTCAGATG			
*Calbindin*	Ileum	F:CATGGATGGGAAGGAGC	62	NM_205513.1	(110)
		R: GCTGCTGGCACCTAAAG			
*Gastrotropin*	Ileum	F:TAGTCACCGAGGTGGTG	60	NM_001277701.1	(110)
		R: GCTTTCCTCCAGAAATCTC			
*MUC2*	Ileum	F:CTGTTGTGGATGGGCGGATTG	62	XM_421035.2	(111)
		R: CCAAACTTGCTGTCCAGCTCC			
*GAPDH*		F: ATGACCACTGTCCATGCCATCCA	56	NM_204305.1	(106)
		R:AGGGATGACTTTCCCTACAGCGTT			

### 2.9 Economic studies

#### 2.9.1 Economic efficiency measures

The economic efficiency parameters examined included cost, return, and net profit. Cost parameters were divided into total variable cost (TVC), and total fixed cost (TFC), and the sum of both TVC and TFC is defined as total cost (TC) (34). The price of purchased broiler chicks and feed costs were considered TVC. The TFC included labor costs, veterinary care costs (vaccines, medications, and veterinary supervision), litter, electricity, water, and miscellaneous costs (35). Additionally, the cost of rent was also included without any modifications. The total return (TR) was determined by adding the sales of both litters and final body weights (36). The net profit (NP) was computed by subtracting from TR and TC (37).

#### 2.9.2 Production and net profit functions

The logarithmic form was the best one to estimate the functions (38).

##### 2.9.2.1 Production function

It is used to determine the relationship between body weights as a dependent variable and feed amount with stocking density as an independent variable.

##### 2.9.2.2 Net profit function

It is used to determine the relationship between net profit as the dependent variable and independent variables such as feed cost, marketing body weights, and stocking density.

### 2.10 Statistical analysis

The data obtained were organized, summarized, and subjected to the one-way ANOVA using IBM SPSS Ver. 25 (39). Before analysis, measurements from multiple broilers within each replicate were averaged, so each replicate had a single average value for each parameter of interest. The experimental data were expressed as “mean ± standard error,” and analyzed using one-way multivariate analysis of variance (MANOVA) and Linear Mixed Models (LMM) in the SAS program to assess the effect of different stocking densities (LSD, MSD, HSD) on various parameters in broiler chickens. Dependent variables included performance, carcass traits, meat quality, behavior, hematological and biochemical markers, oxidative stress indicators, gene expression, and economic parameters. The LMM included fixed effects for stocking density and random effects to account for unit variability, with a random intercept for each unit.

Before conducting MANOVA, the assumptions of normality (Shapiro–Wilk test), homogeneity of variance–covariance matrices (Levene’s test), and equality of covariance matrices (Box’s M test) were tested. Follow-up MANOVAs were conducted to examine the effect of stocking density on each dependent variable separately. Duncan’s New Multiple Range Test (40) was employed to compare means when a significant difference was detected at a confidence level of 95% (*p* < 0.05).

The MANOVA model used was as follows: *Yij* = *μ* + *Aj* + (1∣*Unit_i_*) + *ϵij* where *Yij* represents the vector of observational data for the dependent variables, *μ* is the overall mean vector, *Aj* is the effect of different stocking density levels, and *ϵij* is the vector of random errors.

Results were demonstrated in tables as mean ± SEM. The used formula to determine the percentage of change in productive and economic results is expressed as follows: A percentage of change = [(New Value − Original Value)/Original Value] *100 (41).

## 3 Results

### 3.1 Growth performance

The productivity performance indicators are presented in Table 2. Birds raised at a density of 14 (LSD) and 18 birds (MSD)/m^2^ showed a higher level of productivity as compared to a density of 22/m^2^ (HSD). Elevating the stocking density from 28 to 44 kg/m^2^ significantly decreased body weight. The HSD group exhibited the lowest body weight in comparison to the LSD and MSD groups. At the 6th week of age, it was observed that it was 13.23% lighter than LSD and 5.45% lighter than MSD. The LSD and MSD groups exhibited higher average daily weight gain (ADG) as compared to HSD, which significantly increased by 5.11 and 4.26 g between the 1st and 2nd weeks [*F*_(2, 15) =_ 12.90, *p* = 0.001] versus 18.07 and 27.08 g between the 4th week and 5th week [*F*_(2, 15)_ = 6.09, *p* = 0.012], respectively. Fascinatingly, the period between the 5th and 6th weeks displayed an increase in ADG of LSD and HSD than MSD by 23.54 and 18.16 g, respectively. Concerning relative growth rate (RGR), HSD brings new insights that exhibited the highest values compared to other studied groups. Considering the periods of the 1st–6th week of bird age (Table 3), FI [*F*_(2, 15)_ = 28.79, *p* = 0.0001], BWG [*F*_(2, 15)_ = 8.81, *p* = 0.003], ADG [*F*_(2, 15)_ = 8.80, *p* = 0.003], EPEF [*F*_(2, 15)_ = 8.88, *p* = 0.003], and EBI [*F*_(2, 15)_ = 8.64, *p* = 0.003], were considerably lower in birds reared at high SD than those reared at low and medium SD. Regarding feed intake, LSD consumed 640 g and 869 g more feed than MSD and HSD, respectively. However, the total FCR decreased as the stocking density increased from 28 to 44 kg/m^2^ (Table 2). The feed conversion ratio was higher in the HSD (1.68) and MSD (1.70) groups in comparison to the LSD group (1.81). HSD significantly decreased the BWG (325 g and 105 g) and ADG (9 g and 3 g) compared to LSD and MSD, respectively. This result is due to fewer birds confined in the cages, resulting in increased comfort, more space, and improved accessibility to water and food. The European Broiler Index (EBI) and the European Production Efficiency Factor (EPEF) were also used to assess the performance of broiler chickens. The assessment of the production efficiency using EPEF revealed that the LSD treatment achieved the highest result, which was significantly [*F*_(2, 15)_ = 8.88, *p* = 0.003] greater than the results of the MSD and HSD groups, by 11.16 and 23.82%, respectively.

**Table 2:** Growth performance of broilers raised under various stocking densities from the 1st week.

Productive parameters		Stocking density groups (M ± SE)			*p*-value	*F*-value
		LSD_14_	MSD_18_	HSD_22_		
Body weight (g)	1st week	154.05 ± 6.50^a^	154.54 ± 0.66^a^	125.59 ± 2.41^b^	0.000	17. 00
	2nd week	388.81 ± 12.29^a^	383.33 ± 3.58^a^	324.63 ± 8.52^b^	0.000	16.06
	3rd week	825.15 ± 26.13	820.95 ± 37.87	732.96 ± 41.62	0.156	2.11
	4th week	1437.62 ± 44.80^a^	1319.70 ± 25.82^b^	1248.52 ± 26.02^b^	0.004	8.17
	5th week	1923.33 ± 72.41^a^	1868.48 ± 52.06^a^	1607.78 ± 30.86^b^	0.002	9.58
	6th week	2670.24 ± 88.98^a^	2450.61 ± 43.13^b^	2317.04 ± 26.58^b^	0.003	9.101
Average daily gain (g)	1st–2nd week	33.54 ± 0.84^a^	32.69 ± 0.43^a^	28.43 ± 0.92^b^	0.001	12.90
	2nd–3rd week	62.33 ± 3.74	62.51 ± 5.92	58.33 ± 5.02	0. 800	0.226
	3rd–4th week	87.50 ± 5.28^a^	71.24 ± 1.80^b^	73.65 ± 6.15^ab^	0.063	3.35
	4th–5th week	69.39 ± 3.96^a^	78.40 ± 4.20^a^	51.32 ± 7.77^b^	0.012	6.09
	5th–6th week	106.70 ± 2.37^a^	83.16 ± 2.68^b^	101.32 ± 1.60^a^	0.000	29.75
Relative growth rate	1st–2nd week	86.65 ± 0.99^ab^	85.05 ± 0.45^b^	88.33 ± 0.99^a^	0.049	3.72
	2nd–3rd week	72.15 ± 2.56	72.68 ± 4.28	87.87 ± 10.43	0.201	1.79
	3rd–4th week	54.87 ± 2.80	46.20 ± 2.67	52.50 ± 4.99	0.378	1.04
	4th–5th week	28.79 ± 0.69^ab^	34.32 ± 1.12^a^	25.13 ± 3.79^b^	0.041	3.99
	5th–6th week	32.59 ± 0.44^b^	27.07 ± 1.30^c^	36.19 ± 0.93^a^	0.000	22.99

LSD, low stocking density (14 birds/m^2^); MSD, medium stocking density (18 birds/m^2^); HSD, high stocking density (22 birds/m^2^). Relative growth rate (%) was evaluated according to (112). ^a,b,c^Means with varying superscript letters within the same row exhibited statistically significant differences at *p* < 0.05.

**Table 3:** Growth performance of broilers raised under various stocking densities for all periods (1st week to 6th week) (*n* = 324).

Parameters	Stocking density groups (M ± SE)				
	LSD_14_	MSD_18_	HSD_22_	*P*-value	*F*-value
Feed intake (kg)	4.545.38 ± 0.104^a^	3.905 ± 0.049^b^	3.676.08 ± 0.089^b^	0.000	28.79
BWG (kg)	2.516.19 ± 0.087^a^	2.296.06 ± 0.043^b^	2.191.45 ± 0.255^b^	0.003	8.81
ADG (g)	71.89 ± 2.36^a^	65.60 ± 1.24^b^	62.61 ± 0.73^b^	0.003	8.80
FCR	1.81 ± 0.02^a^	1.70 ± 0.013^b^	1.68 ± 0.019^b^	0.000	23.79
EPEF	352.14 ± 15.21^a^	312.85 ± 16.37^a^	268.25 ± 9.78^b^	0.003	8.88
EBI	398.17 ± 17. 01^a^	351.77 ± 18. 72^ab^	304.45 ± 11.05^b^	0.003	8.64

LSD, low stocking density (14 birds/m^2^); MSD, medium stocking density (18 birds/m^2^); HSD, high stocking density (22 birds/m^2^). BWG, body weight gain; ADG, average daily gain; FCR, feed conversion ratio; EPEF, European production efficiency factor; EBI, European broiler index. ^a,b,c^Means with varying superscript letters within the same row exhibited statistically significant differences at *p* < 0.05.

### 3.2 Carcass characteristics and meat quality

Stocking density had a significant impact on the carcass features. Among LSD, MSD, and HSD birds, the HSD birds exhibited the lowest values for carcass weight (g), breast and thigh weight (g), and dressing percentage (%). The internal organs of the LSD boilers exhibited the highest weight (Table 4). The dressing percentage of LSD was significantly (*F*_(2, 51)_ = 36.00, *p* = 0.0001) higher, at approximately 3 and 4%, compared to MSD and HSD, respectively. The cooking loss of the breast muscle did not exhibit any variations among the different SD treatments. Significant differences were observed only in drip loss [*F*_(2, 51)_ = 8.71, *p* = 0.0001] and pH [*F*_(2, 51)_ = 23.83, *p* = 0.0001], with the maximum drip loss and minimum pH values observed in the HSD group. The stocking density had no discernible effect on the percentage of cooking loss. However, compared to the other groups, CL% tended to be lower in the LSD (Table 5). Conversely, there was a substantial increase in the HSD group in the drip loss [*F*_(2, 51)_ = 8.41, *p* = 0.0001] and cooking loss [F_(2, 51)_ = 18.37, *p* = 0.0001] of thigh muscles with a significantly lower pH [*F*_(2, 51)_ = 143.6, *p* = 0.0001] when compared with LSD and MSD (Table 6).

**Table 4:** Carcass characteristics and yield (% carcass weight) of Avian 48 reared with different stocking densities.

Parameters	Stocking density groups (M ± SE)			*P*-value	*F*-value
	LSD_14_	MSD_18_	HSD_22_		
Live (finishing) weight (kg)	3.050 ± 0.07^a^	2.820 ± 0.04^b^	2.370 ± 0.04^c^	0.000	46.08
Hot carcass weight (kg)	2.250 ± 0.07^a^	2.005 ± 0.03^b^	1.655 ± 0.03^c^	0.000	43.37
Breast weight (g)	1,205 ± 22.77^a^	1,195 ± 26.73^a^	880 ± 13.86^b^	0.000	71.89
Thigh weight (g)	925 ± 20.79^a^	810 ± 7.92^b^	770 ± 5.94^c^	0.000	36.62
Liver weight (g)	68 ± 0.20^a^	65 ± 0.20^b^	53 ± 0.40^c^	0.000	803.3
Heart weight (g)	16.67 ± 0.30^a^	16 ± 0.20^a^	15 ± 0.40^b^	0.002	7.341
Gizzard weight (g)	27 ± 0.20^a^	25 ± 0.99^b^	20.67 ± 0.11^c^	0.000	30.45
Dressing percentage (%)	73.59 ± 0.47^a^	71.04 ± 0.28^b^	69.84 ± 0.04^c^	0.000	36.00

LSD, low stocking density (14 birds/ m^2^); MSD, medium stocking density (18 birds/ m^2^); HSD, high stocking density (22 birds/m^2^). ^a,b,c^Means within the same row with different superscript letters were significantly different.

**Table 5:** Meat quality of breast muscle of broilers reared with different stocking densities.

Parameters	Stocking density groups (M ± SE)			*P*-value	*F*-value
	LSD14	MSD18	HSD22		
pH	5.82 ± 0.02^a^	5.95 ± 0.03^b^	5.76 ± 0.01^c^	0.000	23.83
Drip loss %	8.00 ± 0.56^a^	10.50 ± 0.61^b^	13.00 ± 1.21^c^	0.000	8.713
Cooking loss %	17.96 ± 0.1	18.79 ± 0.44	18.79 ± 0.44	0.188	1.729

LSD, low stocking density (14 birds/m^2^); MSD, medium stocking density (18 birds/m^2^); HSD, high stocking density (22 birds/m^2^). ^a,b,c^Means within the same row with different superscript letters were significantly different.

**Table 6:** Meat quality of thigh muscle of broilers reared with different stocking densities.

Parameters	Stocking density groups (M ± SE)			*P*-value	*F*-value
	LSD14	MSD18	HSD22		
pH	6.27 ± 0.02^a^	6.25 ± 0.03^a^	5.99 ± 0.01^b^	0.000	143.6
Drip loss %	11.50 ± 0.85^a^	8.00 ± 0.40^b^	13.00 ± 1.21^a^	0.000	8.411
Cooking loss %	17.25 ± 0.27^b^	17.82 ± 0.04^b^	19.11 ± 0.27^a^	0.000	18.37

LSD, low stocking density (14 birds/m^2^); MSD, medium stocking density (18 birds/m^2^); HSD, high stocking density (22 birds/m^2^). ^a,b,c^Means within the same row with different superscript letters were significantly different.

### 3.3 Behavior and fear reactions

#### 3.3.1 Tonic immobility test

**Figure 1:**
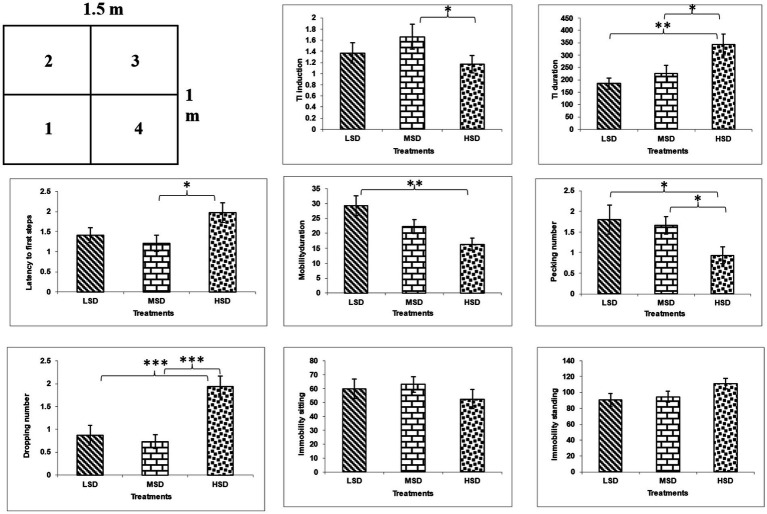
Open field test arena (1.5×1 m), with the arrow indicating the starting point of the chick during the test and behavioral results of TI and OFT. *P ≤ 0.5, **P ≤ 0.01, and ***P ≤ 0.001.

Regarding behavior and fear reactions, stocking density had a significant impact on TI induction [*F*_(3, 42)_ = 18.028, *p* = 0.0001] as shown in (Figure 1). The birds in the HSD group showed the significantly lowest TI induction, followed by birds in LSD and MSD. Additionally, the stocking density had substantial effects on broiler TI duration [*F*_(3, 42)_ = 5.693, *p* = 0.006], with the birds in the LSD group showing lower TI duration than those in the MSD and HSD groups.

#### 3.3.2 Open field test

The boilers reared under different stocking densities significantly affected their behavior in the open field test (Figure 1) as latency to first step [*F*_(3, 42)_ = 3.385, *p* = 0.043], mobility duration [*F*_(3, 42)_ = 6.22, *p* = 0.004], immobility duration [*F*_(3, 42)_ = 6.22, *p* = 0.004], pecking [*F*_(3, 42)_ = 3.072, *p* = 0.05], and droppings [*F*_(3, 42)_ = 10.646, *p* = 0.0001]. The birds in the densely populated area exhibited the highest values in latency to the first step, duration of immobility, and number of droppings. However, they exhibited the lowest values in duration of mobility and pecking compared to those in LSD and MSD.

### 3.4 Hematological, biochemical, and oxidative stress parameters

Regarding how stocking density affects hematological indicators (Table 7), SD groups’ Hb values differed significantly. Hb and RBCs were significantly [*F*_(2, 51) =_ 6.16, *p* = 0.011] and [*F*_(2, 51)_ = 23.57, *p* = 0.0001, respectively] increased in the HSD group as compared to the LSD and MSD groups. Similarly, heterophils were significantly [*F*_(2, 51)_ = 23.77, *p* = 0.0001] increased in the HSD group compared to the LSD and MSD groups. In addition, no significant difference was found in eosinophils and monocyte count among the groups under study. Conversely, data in Table 8 demonstrate that cortisol markedly [*F*_(2, 51)_ = 19.02, *p* = 0.0001] decreased in the LSD group as compared to the MSD and HSD groups. TAC was significantly higher in the LSD and MSD groups as compared to the HSD group; however, the MDA levels did not significantly differ among the three groups. To assess the effect of stocking density on immunity, IgG was measured, which displayed a significantly [*F*_(2, 51) =_ 5.91, *p* = 0.013] lesser value in the MSD and HSD groups than in the LSD groups. The study found a significant impact of SD on total protein [*F*_(2, 51) =_ 17.16 *p* = 0.0001], albumin [*F*_(2, 51)_ = 8.96, *p* = 0.003], globulin [*F*_(3, 42)_ = 8.75, *p* = 0.003], and thyroxine hormone (T4) [*F*_(2, 51)_ = 8.96, *p* = 0.001]. The MSD and HSD groups showed higher levels of these parameters compared to the LSD group.

**Table 7:** Effect of stocking density on Hb, RBCs, and total and differential WBC count.

Parameters	Stocking density groups (M ± SE)			*P*-value	*F*-value
	LSD_14_	MSD_18_	HSD_22_		
Hb	10.00 ± 0.48^b^	10.43 ± 0.50^b^	12.80 ± 0.79^a^	0.011	6.163
RBCs	3.54 ± 0.18^b^	3.47 ± 0.04^b^	4.80 ± 0.19^a^	0.000	23.57
WBCs	16.83 ± 1.26	13.4 ± 0.40	17.13 ± 1.69	0.092	2.814
Heterophils	14.67 ± 0.76^c^	18.00 ± 1.32^b^	25.00 ± 1.1^a^	0.000	23.77
Lymphocytes	81.00 ± 0.97^a^	78.67 ± 0.84^a^	70.67 ± 1.52^b^	0.000	22.28
Eosinophils	0.33 ± 0.21	0.33 ± 0.21	0.67 ± 0.21	0.454	0.833
Monocyte	4.00 ± 0.37	3.00 ± 0.37	3.67 ± 0.42	0.207	1.750

^a,b,cMeans within the same row with different superscript letters were significantly different.^

**Table 8:** Effect of stocking density on indicators of biochemical and oxidative stress.

Parameters	Stocking density groups (M ± SE)			*P*-value	*F*-value
	LSD_14_	MSD_18_	HSD_22_		
Cortisol	0.47 ± 0.08^b^	0.80 ± 0.04^a^	0.97 ± 0.06^a^	0.000	19.02
MDA	43.63 ± 2.66	42.87 ± 1.45	51.7 ± 5.25	0.176	1.955
TAC	0.74 ± 0.09^a^	0.73 ± 0.07^a^	0.43 ± 0.03^b^	0.007	6.922
IgG	11.00 ± 0.37^a^	8.33 ± 0.56^b^	9.00 ± 0.73^b^	0.013	5.909
Total protein	2.57 ± 0.08^b^	3.00 ± 0.04^a^	3.07 ± 0.08^a^	0.000	17.16
Albumin	1.33 ± 0.04^b^	1.57 ± 0.06^a^	1.53 ± 0.02^a^	0.003	8.958
Globulin	1.23 ± 0.0^b^	1.43 ± 0.04^a^	1.53 ± 0.06^a^	0.003	8.750
T4	0.90 ± 0.04^c^	1.03 ± 0.02^b^	1.17 ± 0.06^a^	0.001	8.960

MDA, malondialdehyde; TAC, total antioxidant capacity; T4, thyroxine hormone. ^a,b,c^Means within the same row with different superscript letters were significantly different.

### 3.5 Gene expression of immune and growth markers

Birds of LSD showed a notable up-regulation of growth (IGF1 and GH), as shown in Figures 2 and 3, intestinal health (Muc2, Cath-B, *Calbindin*, and *Gastrotropin*), as demonstrated in Figures 4–7, and anti-inflammatory (IL-10) genes (Figure 8) compared to MSD and HSD. Meanwhile, inflammatory (IL-6, IL-8, and IL-13) markers were significantly down-regulated (Figures 9–11).

**Figure 2:**
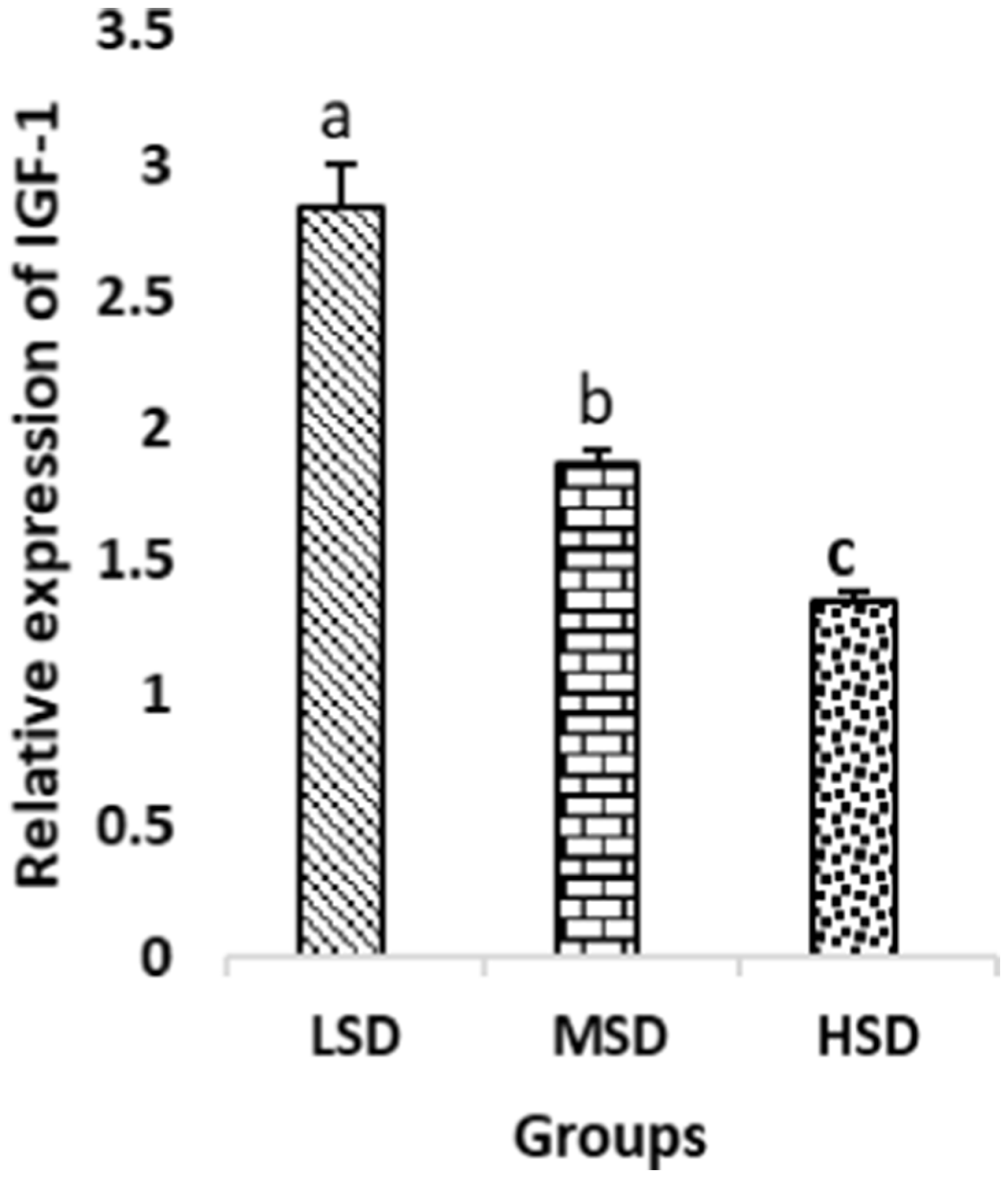
Relative expression of IGF-1. Different superscript letters for were significantly different (*P* < 0.05).

**Figure 3:**
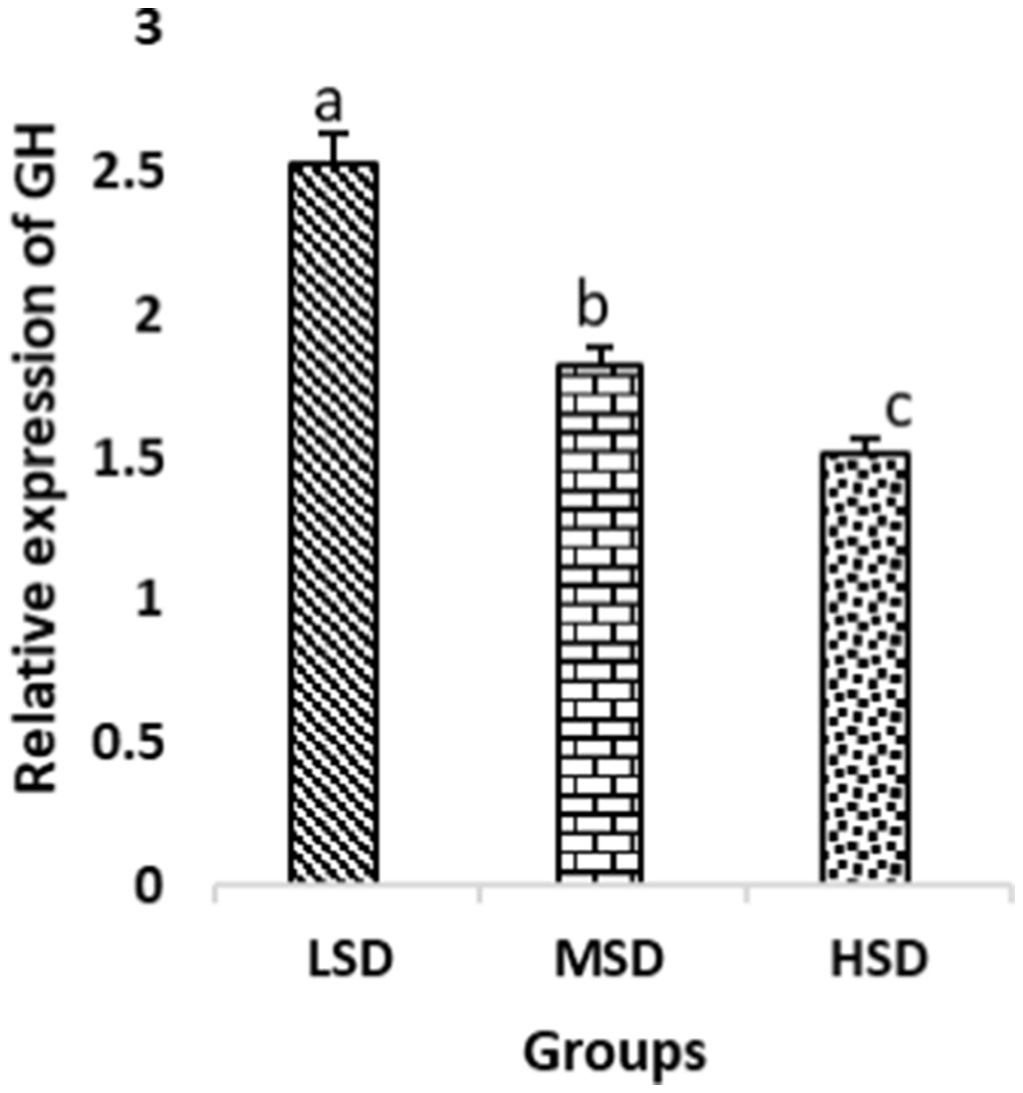
Relative expression of GH. Different superscript letters for were significantly different (*P* < 0.05).

**Figure 4:**
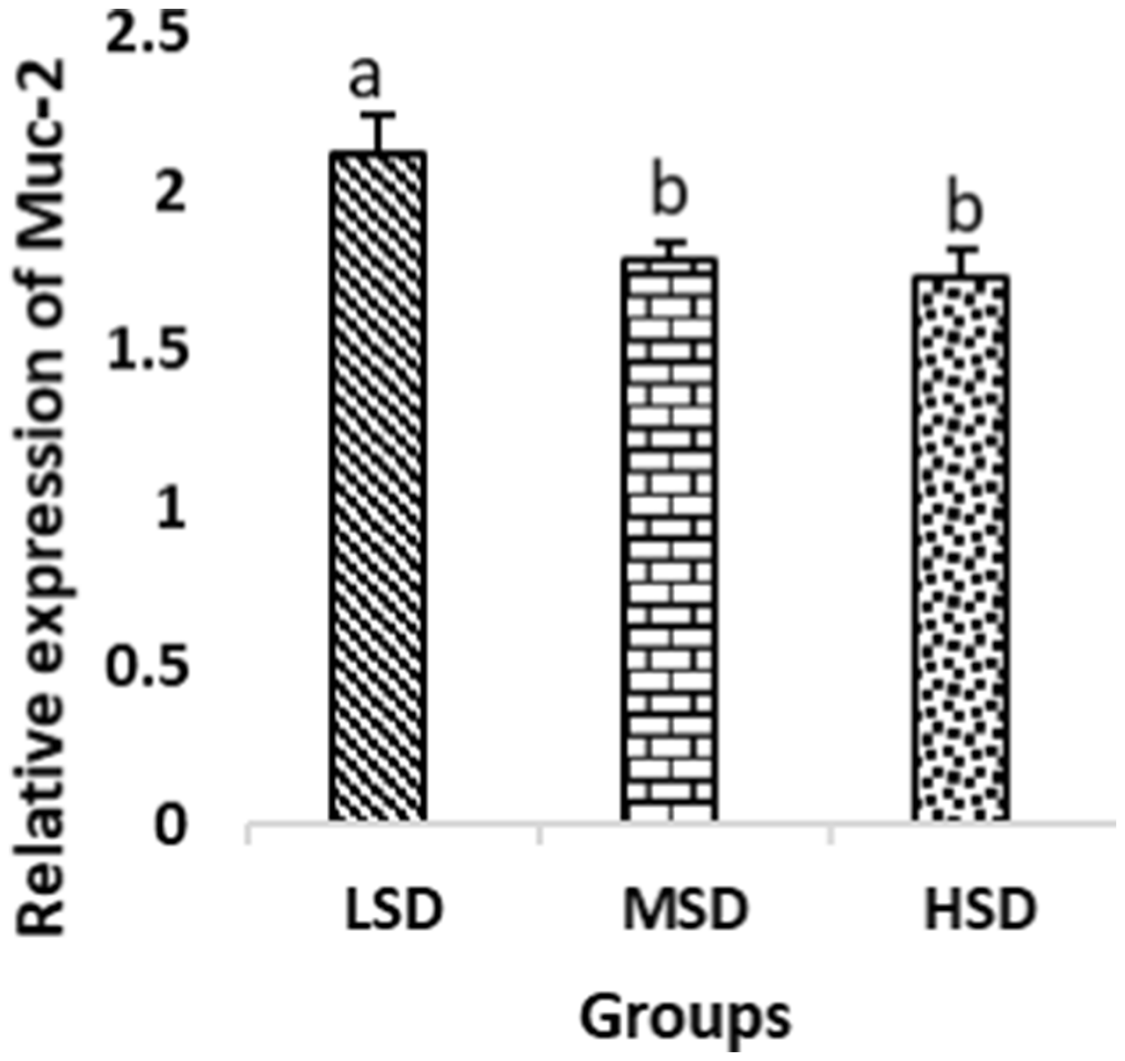
Relative expression of Muc-2. Different superscript letters for were significantly different (*P* < 0.05).

**Figure 5:**
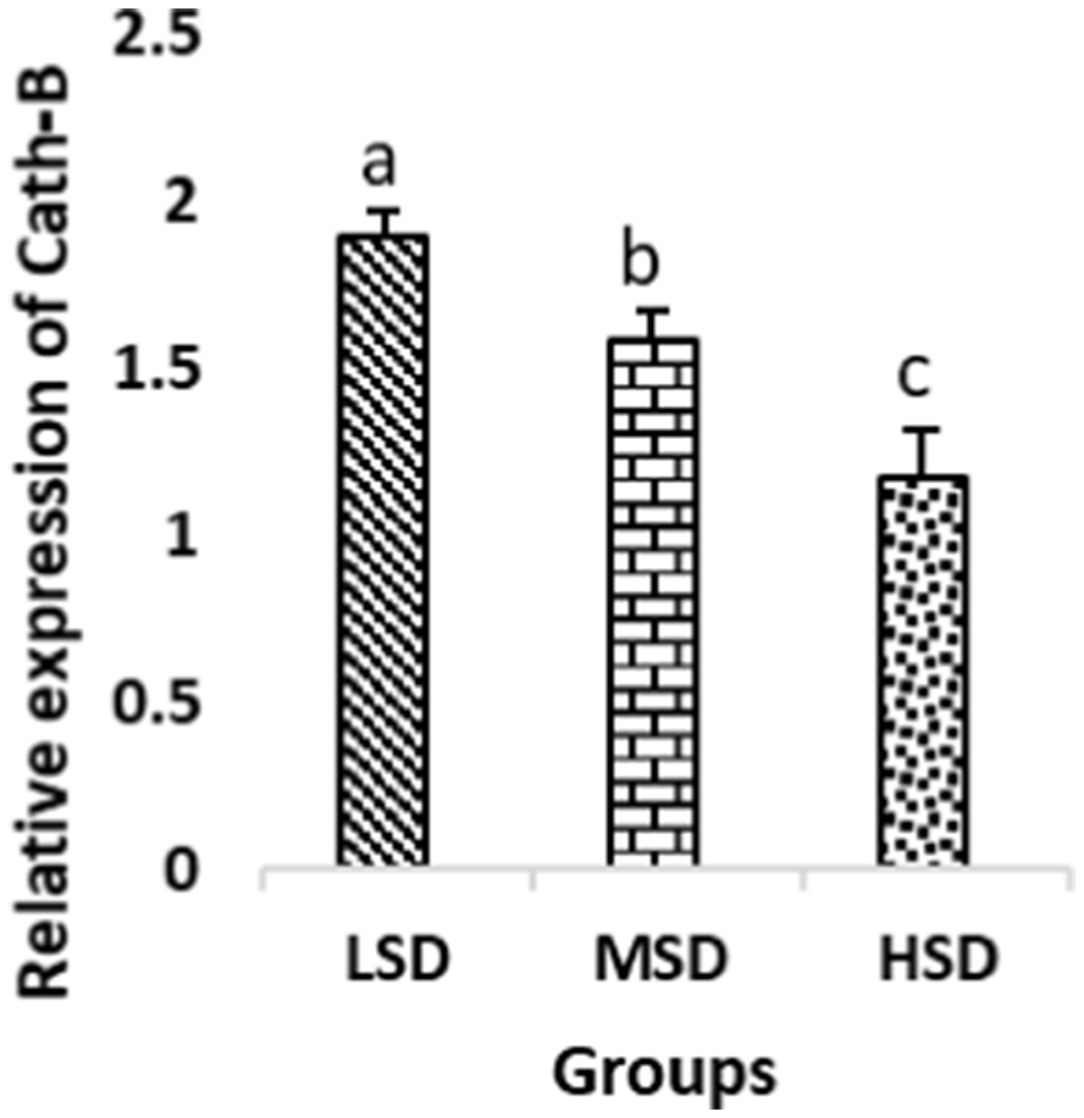
Relative expression of Cath-B. Different superscript letters for were significantly different (*P* < 0.05).

**Figure 6:**
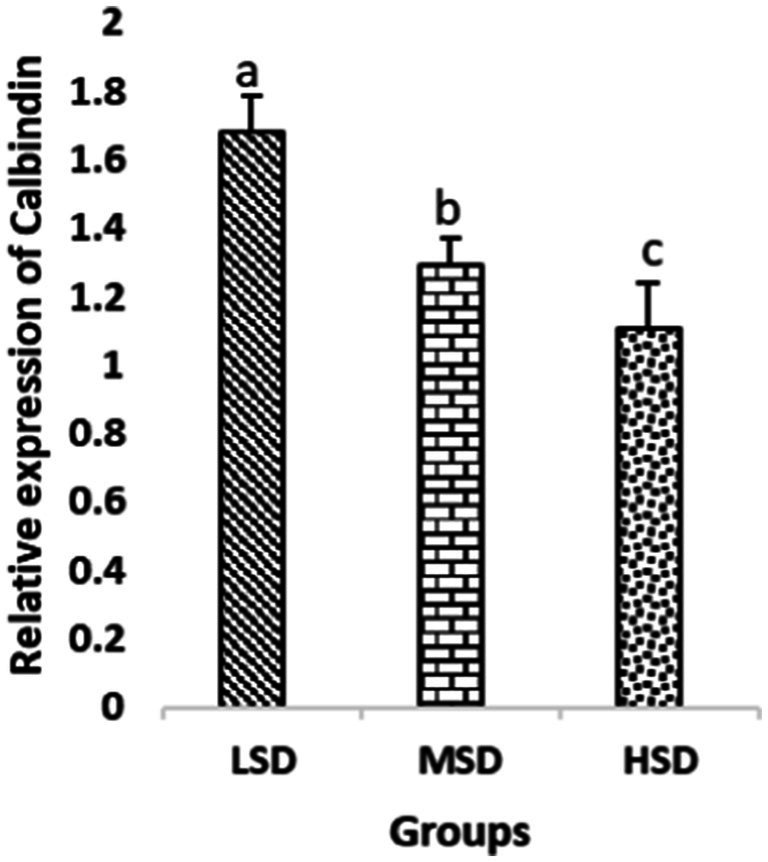
Relative expression of *Calbindin*. Different superscript letters for were significantly different (*P* < 0.05).

**Figure 7:**
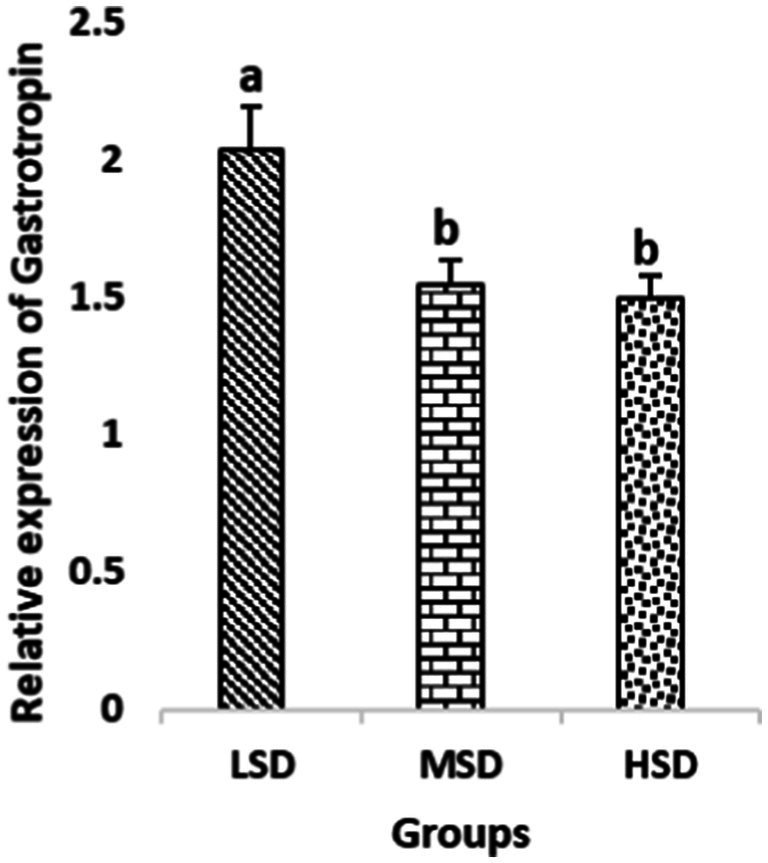
Relative expression of *Gastrotropin*. Different superscript letters for were significantly different (*P* < 0.05).

**Figure 8:**
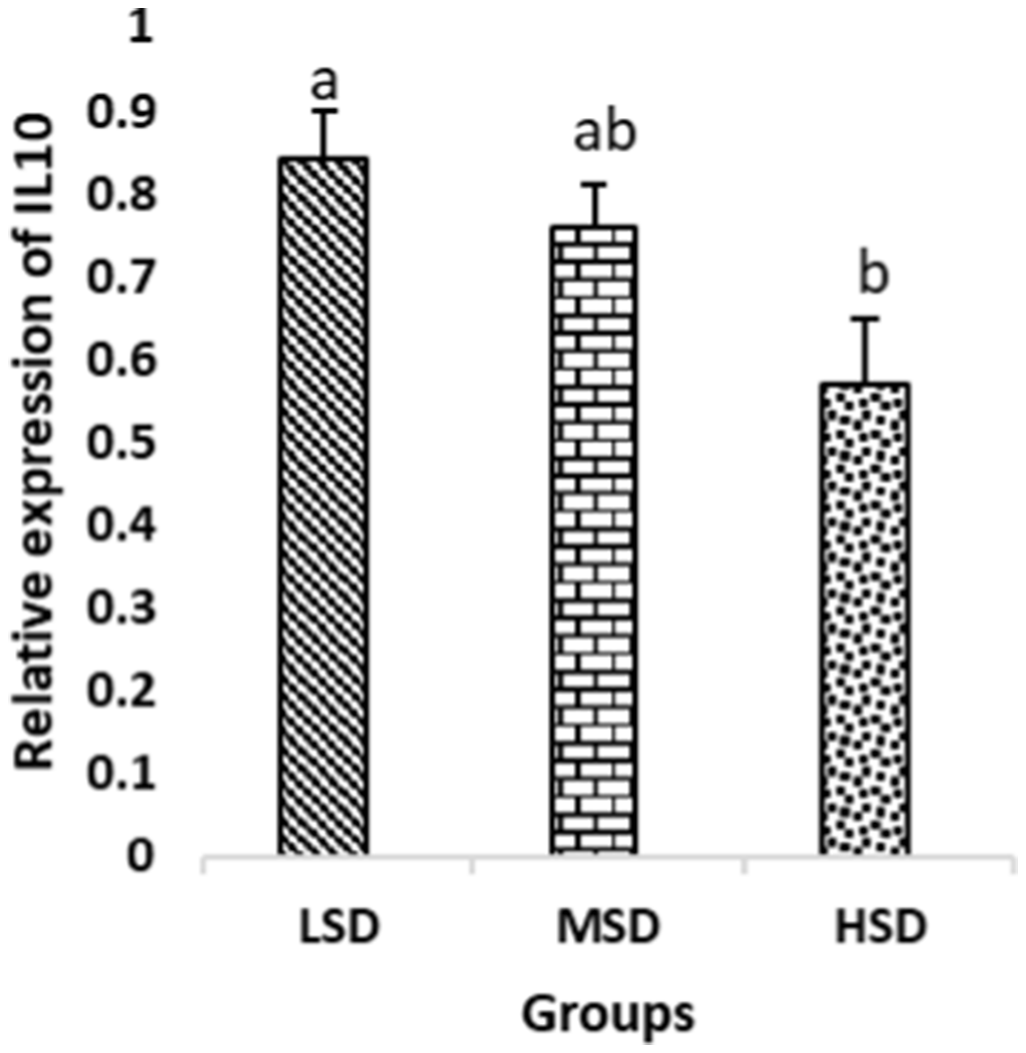
Relative expression of IL10. Different superscript letters for were significantly different (*P* < 0.05).

**Figure 9:**
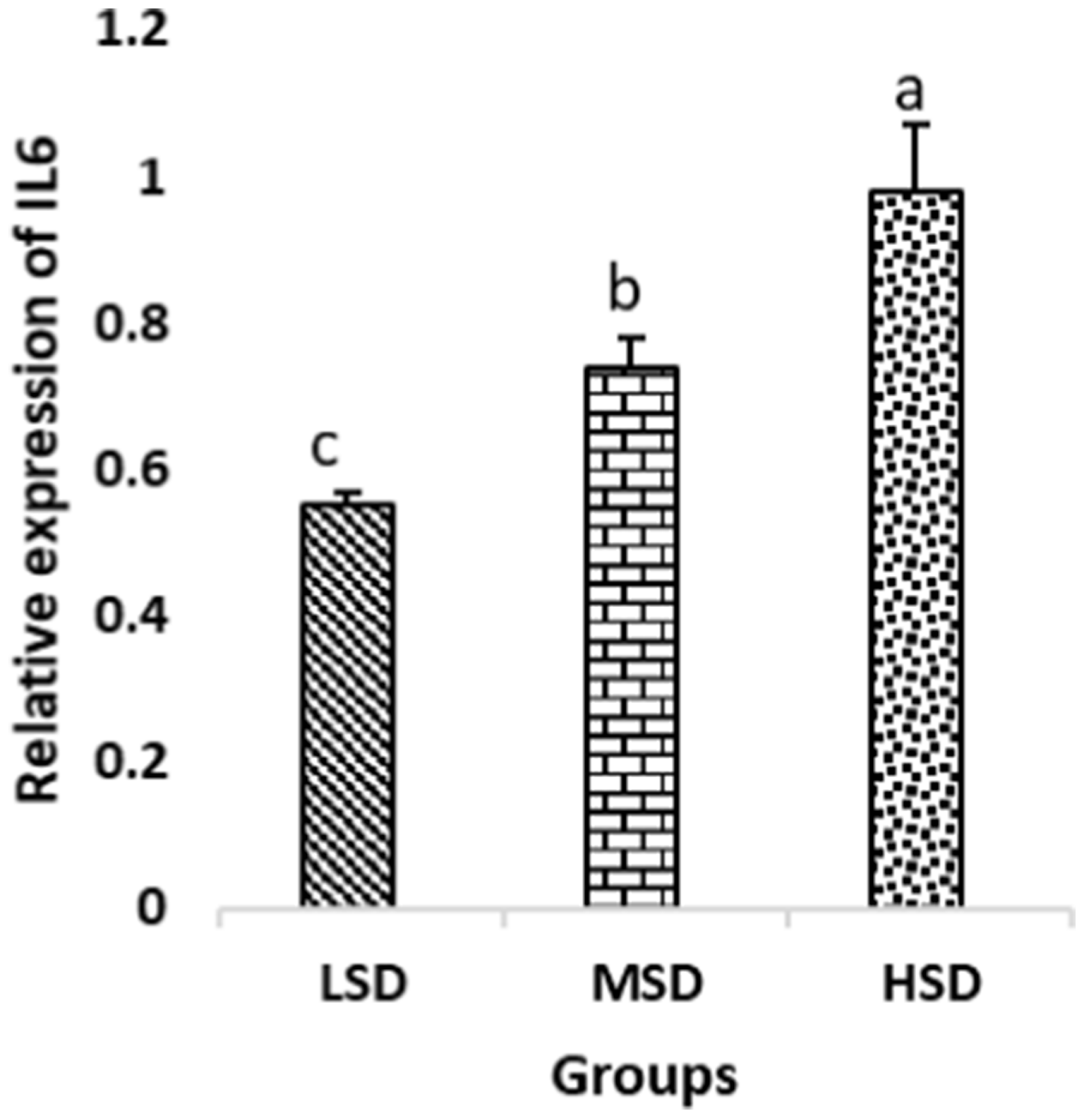
Relative expression of IL6. Different superscript letters for were significantly different (*P* < 0.05).

**Figure 10:**
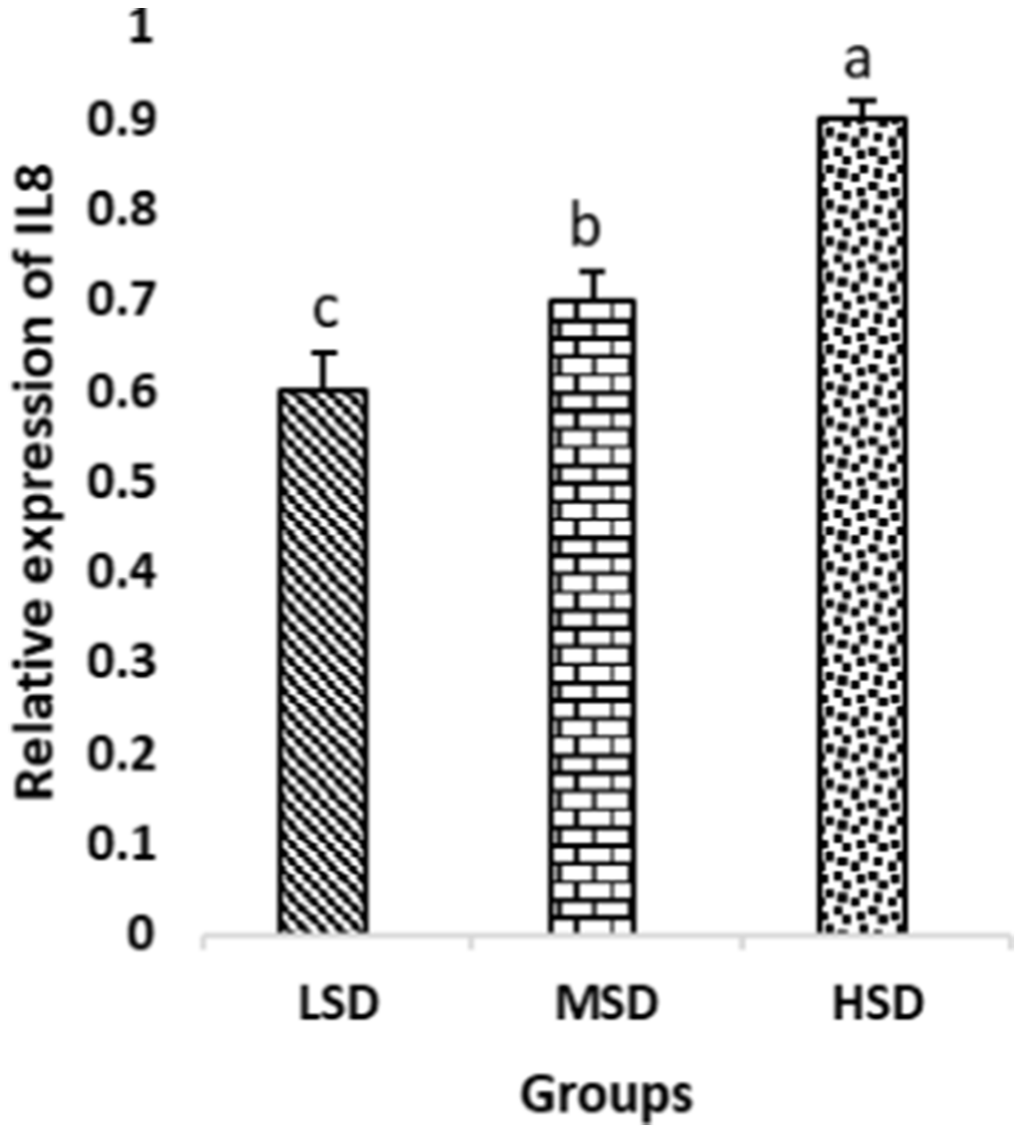
Relative expression of IL8. Different superscript letters for were significantly different (*P* < 0.05).

**Figure 11:**
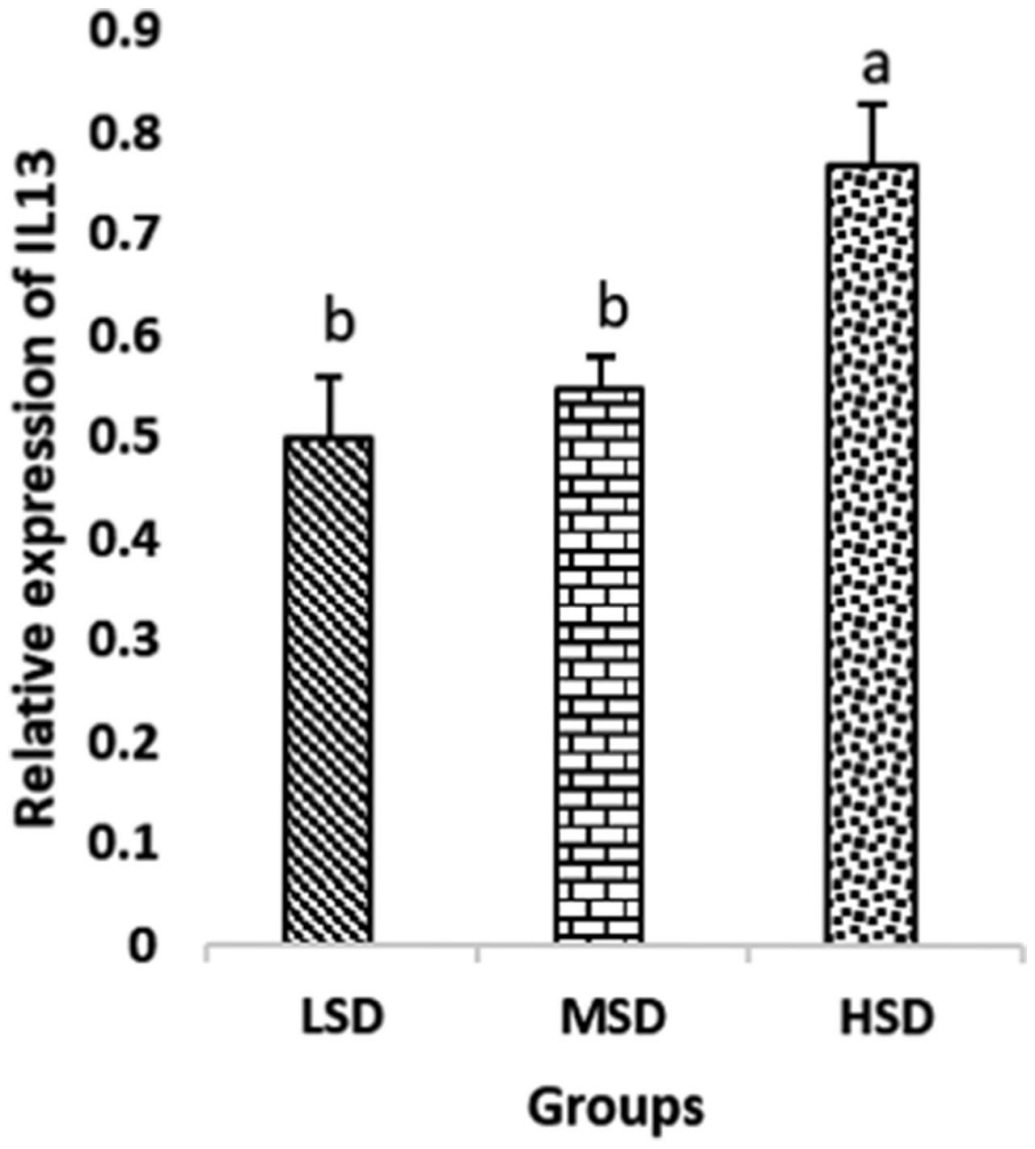
Relative expression of IL13. Different superscript letters for were significantly different (*P* < 0.05).

### 3.6 Economic parameters

Data shown in Table 9 presents the economic parameters of three groups of different stocking densities. Our study indicated that there were no significant differences in the estimated TFC during the production process within each experimental group. However, there was a significant difference in TFC between the groups (*p* = 0.0001), with the highest value observed in the HSD group. On the contrary, there was a notable variation [*F*_(2, 15)_ = 0.361*, p* = 0.001] in feed cost per group among birds reared at different stocking densities. Birds kept in low-density groups incurred a higher feed cost per bird (40.91 LE) compared to birds from medium and high-density groups (35.15 and 33.08 LE, respectively). Conversely, the groups with high and moderate stocking densities incurred higher feed costs of 727.86 and 632.61 LE, respectively, compared to the low stocking density group, which had a cost of 572.72 LE per group. In the same way, the TVC [*F*_(2, 15)_ = 0.361, *p* = 0.001] and TC [*F*_(2, 15)_ = 0.553, *p* = 0.001] per group showed a significant increase in both HSD (903.8 and 1365.4 LE/ group) and MSD (776.6 and 1161.7 LE/group) compared to LSD (684.72 and 1004.1 LE/ group), respectively. Body weight sales per group were higher significantly [*F*_(2, 15)_ = 0.757, *p* = 0.041], in the HSD group (1349.9 LE/group) compared to the MSD and LSD groups, which were 1157.2 and 1046.7 LE/group, respectively. Conversely, body weight sales per bird were significantly [*F*_(2, 15)_ = 1.13, *p* = 0.021] highest in birds from LSD groups (85.40 LE/bird) as opposed to MSD and HSD at 78.69 and 66.36 LE/bird, respectively. Similarly, the TR group showed a notable distinction at [*F*_(2, 15)_ = 0.783, *p* = 0.001], with a greater level in the HSD group (1474.58 LE/group) compared to the MSD and LSD groups (1433.26 and 1204.92 LE/group), respectively. Conversely, TR/bird was significant at [*F*_(2, 15)_ = 1.13*, p* = 0.021], with a higher level in the LSD group (86.07 LE/bird) than in the MSD and HSD groups (79.63 and 67.03 LE/bird), respectively.

**Table 9:** Effect of different levels of stocking density on economic parameters.

	Stocking density groups (M ± SE)				
Item (LE)	LSD14 (M ± SE)	MSD18 (M ± SE)	HSD22 (M ± SE)	*P*-value	*F*-value
Item/bird					
Chicks’ cost	8	8	8	--	--
Total feed cost	40.91 ± 7.26^a^	35.15 ± 7.18^b^	33.08 ± 15.70^b^	0.001	0.361
TVC	48.91 ± 7.26^a^	43.15 ± 7.18^b^	41.09 ± 15.70^b^	0.001	0.361
TFC	22.81	21.40	20.98	---	--
TC	71.72 ± 7.26^a^	64.54 ± 7.18^b^	62.06 ± 15.70^b^	0.001	0.553
BW sale	85.40 ± 8.73^a^	78.69 ± 10.57^b^	66.36 ± 7.83^c^	0.021	1.13
Litter sales	0.67	0.67	0.67	---	--
TR/bird	86.07 ± 8.73^a^	79.63 ± 10.57^b^	67.03 ± 7.83^c^	0.021	1.13
NP/bird	14.35 ± 1.41^a^	15.09 ± 2.58^a^	4.97 ± 2.78^b^	0.033	0.791
Item/group					
Total chicks’ cost	112	144	176	---	--
Total feed cost	572.72 ± 101.62^C^	632.61 ± 129.21^B^	727.86 ± 125.34^A^	0.0001	0.659
TVC	684.72 ± 101.62^C^	776.61 ± 129.21^B^	903.81 ± 125.34^A^	0.0001	0.447
Litter cost/group	42	83.32	133.32	---	--
Labor cost/group	30	32	34	---	--
Water & electricity cost/group	70	74	80	---	--
Rent cost/group	80	80	80	---	--
Vet. management costs/group	57.4	73.8	90.2	---	--
Miscellaneous costs/group	40	42	44	---	--
TFC	319.40^C^	385.12^B^	461.52^A^	1.33	0.0001
TC	1004.12 ± 101.62^C^	1161.73 ± 129.21^B^	1365.33 ± 125.34^A^	0.001	2.302
BW sales	1195.60 ± 122.23^B^	1421.28 ± 190.31^A^	1459.92 ± 172.19^A^	0.041	0.757
Litter sales	9.32	11.98	14.66	---	--
TR	1204.92 ± 299.40^B^	1433.26 ± 466.16^A^	1474.58 ± 421.77^A^	0.001	0.783
NP/group	200.8 ± 19.70A^B^	271.53 ± 46.40^A^	109.25 ± 61.14^B^	0.041	0.857

Production regression function			
Independent variables	Elasticity measure	*t*-value	*P*-value
Intercept	−1.075	−0.764	1.13
Log feed intake	1.174	3.612	0.008
log stocking density	−0.43	−0.243	0.03

Economic net profit function			
Independent variables	Elasticity measure	*t*-value	*P*-value
Intercept	−67.537	−3.914	0.04
Log marketing BW	30.966	4.113	0.007
log feed cost	−22.970	−3.245	0.02
log stocking density	−7.948	−4.818	0.03

The mean values with different superscript capital letters for groups within the same row were significantly different (*P* < 0.05). The mean values with different superscript small letters for birds within the same row differ significantly at *P* < 0.05. M, mean; SE, standard error; TC, total costs; TVC, total variable costs; TFC, total fixed costs; TR, total returns; NP, net profit; BW, body weight. L.E, Egyptian Pound. *Price of kg broiler sale at the time of the experiment = 28 L.E. (All prices used in the study were equal to market prices at the time of the experiment).

The NP values significantly increased for both group [*F*_(2, 15)_ = 0.857, *p* = 0.041], and individual bird [*F*_(2, 15)_ = 0.791, *p* = 0.033] measurements in MSD (271.53 LE/group and 15.09 LE/bird) and LSD (200.8/group and 14.35 LE/bird) and compared to HSD (109.25 LE/group and 4. 97 LE/bird).

### 3.7 Productive and economic function

The results of the logarithmic production function in Table 9 demonstrate that the function is significant at *p* < 0.05 and *p* < 0.01. The analysis of the function reveals that the factors influencing production (marketing body weight) in broiler farms (such as feed quantity and stocking density) account for approximately 90% of the variations observed (R̅^2^ = 0.90). The findings showed that the mean elasticity of feed quantity was approximately 1.17, signifying that a 1% increase in feed amount would result in around a 1.17% rise in production. This outcome confirmed the positive impact of feed amount on production. The results also indicated that the average elasticity of stock density was about −0.43, suggesting that a 1% increase in stock density would lead to a decrease of approximately −0.43% in production. The NP function shown in Table 9 is statistically significant at (*p* < 0.05 and *p* < 0.01), revealing a positive impact of marketing body weight and an adverse impact of both feed cost and stocking density on NP. The economic function of net profit indicates that the marketing body weight sales, feed costs, and stocking density account for approximately 90% of the fluctuations in broiler farm net profit (R̅^2^ = 0.90). The average elasticity of the marketing body weight sales was around (30.966), suggesting that a 1% increase in marketing body weight sales would lead to an approximately 30.966% rise in NP.

## 4 Discussion

The current investigation examined the potential consequences of various stocking densities on the performance, carcass characteristics, hematological and biochemical parameters, immune response, oxidative stress indicators, behavioral and welfare evaluations, gene expression profiles of immune and growth markers, and economic parameters of Avian 48 broiler breeds reared under different stocking densities. The aim was to determine the optimal stocking density to provide better welfare conditions; lower production cost and improves quality. The reasons behind negative outcomes (reduction of growth performance) from high SD has been correlated with numerous environmental circumstances as declined gaseous and thermal exchange in the birds’ microenvironment (42), increased coccidiosis in broilers (43), disturbance in the digestive microbiota that both causes intestinal impairment and restrict both the digestion and absorption of nutrients that may decrease the performance of broiler, difficulty accessing feed and water under HSD because of competition between birds and restraining of the movement of birds inside a floor area (44, 45), increased dust and airborne pathogens may also affect performance negatively at HSD (46), reduced ability to dissipate body heat to the surroundings and so consumed less feed to maintain their homeothermic state (47), increased hypothalamic MC4R mRNA expression resulting in decreasing feed intake (48), decreased intestinal barrier function (by elevating oxidative stress and inflammatory reactions) might be one of the causes of the poor performance of broiler chickens reared at HSD (44).

The growth of poultry is a quantitative characteristic regulated by nutrition, environment, and genetics. Variations in growth performance can be linked to the negative consequences of dense stocking and the interplay between environment and genetics (49), which have been confirmed by several authors (50). However, other researchers found no impact of stocking density on growth performance (51). Our findings were consistent with other research indicating that growth performance was negatively impacted by increased stocking density. The best performance was achieved with the LSD (14 birds/m^2^) densities and MSD (18 birds/m^2^), with no significant discrepancies observed. The poorest level of performance was observed at high stocking densities (22 birds/m^2^) due to the increased number of birds per m^2^, which restricted the birds’ freedom of movement and access to feeders and drinkers.

Furthermore, this high density resulted in decreased comfort and bird performance due to bird accumulation and increasing ambient temperatures, primarily due to a decline in feed intake (52), which aligns with our results. Other authors also observed this tendency, who reported reduced total weight gain from 142.38 to 106.52 g as stocking density increased from 14 to 22 chickens/m^2^ at the age of 42 days (45). The decrease in feed intake and weight gain might indicate that the stocking density stress negatively impacted the broiler chickens’ performance (53). The maximum body weight and weight gain were correlated with the minimal stocking density (14 birds/m^2^) and contrariwise for the greatest stocking density (22 birds/m^2^) (52). This trend was similarly observed previously (5), who found that birds with stocking densities of 10 and 20 chicks /m^2^ densities exhibited the greatest and minimal body weights, respectively. In addition, stocking density had a substantial effect on the final body weight, supporting our result. This finding can be explained by two actions that cause the yield to increase: Firstly, it decreases the temperature and raises the bird’s surface ventilation (54). The current findings can be ascribed to the larger feeder space allotted to each bird at 14 chicks per m^2^ density. That gives birds with lower stocking densities greater access to food and water, reducing anxiety levels (55). Ovseychik and Lukashenko (56) reported that at 28 days of age, the rise in live body weight with the lessening in stocking density was found to have a significant effect. Nevertheless, our findings contradict previous findings (57) demonstrated that stocking densities of 10 to 20 birds/ m^2^ did not affect the growth performance and broilers’ weight gains. Furthermore, it did not affect 30 and 40 kg body weight/ m^2^ for Ross 308 and Avian 48, respectively (58, 59), nor 25 to 40 kg/ m^2^ of body weight on d 7 for Cobb 500 (55). Compared to the low SD treatments, birds’ weight gains in the high SD treatments were reduced by 11.9% without a significant effect among the stocking density treatments (53). This result could be due to relatively limited behavioral activities (feeding, drinking, and possibly resting) among the birds due to the high stocking density, which enhanced high weight gain to their feed intake (57). Our results revealed that high stocking density decreases feed intake (58), as confirmed previously by Ha et al. (53), who observed that feed intake was 13.9% lower at high stocking density. Nevertheless, total feed consumption was considerably greater under the highest (40 kg/m^2^) and minimal (25 kg/m^2^) stocking densities than by 35 kg/m^2^ (55). According to the current results regarding FCR, high stocking density (22/ m^2^) attended to get a better feed conversion ratio compared to low (14/ m^2^) and medium (18/m^2^) stocking density that matched with previously published research which recorded that a decrease in stocking density has an adverse effect on feed conversion (6, 60). One probable explanation is that the birds in the LSD group had greater room for movement and feeding accessibility. Consequently, even though they ate more feed, energy losses prevented them from efficiently converting it into tissues (52).

However, published research consistently indicates that if space restrictions are less than 0.0625–0.07 m^2^/bird (34–38 kg/m^2,^ based on the final body weight) or at densities above 19 birds/m^2^ in environmentally controlled broiler houses, resulting in the wellbeing and welfare of broilers is declined. In such cases, chickens exhibit slower growth and have a lesser feed conversion ratio compared to lower densities (19). Furthermore, the ambient air temperature has a significant impact on both growth and feed intake. For instance, in a colder climate, the bird consumes more to produce more energy and warm its body. Conversely, less energy is required to warm the bird’s body due to the reduced ratio of surface area to the number of birds. At this stage, most of the energy obtained from the diet is used to facilitate the growth of the bird’s body. Therefore, the bird’s conversion rates increase (5). Elevated stocking density declined body weight gain and feed intake (45). Under these conditions, FCR remained unaffected in response to SD. Therefore, achieving optimal broiler chicken production in HSD requires effectively managing both HSD and creating a comfortable environment on the farm (61).

Nonetheless, several authors have demonstrated that increased stocking density positively impacts FCR (19, 62). Despite the unfavorable effect of high stocking density on performance, several observers failed to see any differences (21, 22). The European Production Efficiency (EPEF) and the European Broiler Index (EBI) were also used to assess the broiler chickens’ performance. Our results aligned with previous study asserted that the consequences of stocking density on the production index were significant (5).

Moreover, the highest and lowest production indicators were, respectively, at the lowest (10 chicks/m^2^) and highest density (20 chicks/m^2^). Greater values of these variables verify the flock’s wellbeing and the uniformity of its weight gain (63), which confirmed our results. This finding is inconsistent with Kryeziu et al. (52) who indicated no significant differences among groups in EPEF and EBI values according to feed intake. Birds retained at a stocking density of 22 broilers/m^2^ had the least during the experiment, explaining the decreased live body weight.

Previous research validates our findings, which have shown that increased stocking density can have a detrimental effect on various aspects of carcass quality and yield, including carcass yield (52, 53), whole breast yield (16, 53), thigh (22), and dressing percent (16). In contrast, significant improvement was observed in carcass characteristics up to a density of 14 birds/m^2^ in broiler chickens (64). Highly significant differences in the absolute weights of carcasses, giblets, and total edible parts were observed owing to the stocking density effect (61), which confirmed our results. Nevertheless, other studies have suggested that SD does not affect carcass yield (6, 45), breast and thigh yields of broiler chickens (45, 65), internal organs (66), liver weight (53), and dressing percent (60). The result’s cooking loss and drip loss percentages were confirmed before by Li et al. (22) who observed that high stocking density induces oxidative stress, which is one of the primary reasons meat quality is declining. The oxidation of meat can lead to decreased sensitivity to hydrolysis, increased protein degradation, and reduced water-holding density of myofibrils, increasing the water loss of the meat (14), which supports our results.

According to our findings, ultimate muscle pH at HSD exhibited a favorable correlation with LSD and MSD. This finding might be explained by pHu, which has a strong negative correlation with drip loss (67) and cooking loss (68) brought on by a faster lactate deposit and progressively higher meat toughness (69). Meat that has lost some of its quality because of denaturing proteins has a lower glycogen deposit when the meat has a low pH (70), with altered meat quality as a result of denaturing proteins and consequently increased cooking loss (71). Conversely, our result is incompatible with those of other authors who found that the pH and cooking loss of the muscle were all unchanged by different stocking densities (21, 45, 53, 72). It can be argued that breast meat quality may be adversely influenced by HSD’s impact on litter quality (45).

It is no longer appropriate to evaluate an animal’s welfare status using only one measurable indicator. Consequently, to obtain a precise and scientific assessment of an animal’s wellbeing, it is necessary to employ a multidisciplinary approach that incorporates indicators related to production, physiology, and behavior. An external element influencing avian activity, behavior, and wellbeing is stocking density. Raising hens for broiler production at various densities affected their TI reactions and responses in the OF arena. Stocking significantly influenced the tonic immobility (TI) duration, with the shortest duration recorded at the lowest stocking density. Similar results were demonstrated previously (73). This finding could be the result of higher stocking densities, which could lower the wellbeing of broiler hens by causing them to experience negative emotional states. We hypothesize that those birds from high-density pens were more anxious compared to birds from low-density pens.

According to a previous study, high stocking densities have been linked to increased fearfulness in broilers. Anxiety and dread are welfare issues because they can have negative impacts and, if persistently stimulated, might emphasize an animal’s incapacity to adapt to its surroundings. Fear is a transient emotional reaction that drives one to flee from or freeze in the face of an imminent threat to survival. Adverse prenatal and postnatal life events amplify anxiety, a longer-term emotional reaction that drives vigilance (i.e., awareness) in response to a perceived potential threat (74). The primary use of blood biochemical profiles as indicators of the physiological and metabolic state of broilers (75). In the current study, broilers’ blood profiles showed higher RBCs and heterophil-to-lymphocyte ratio levels in the high-density group than in the medium and low-density groups. Our results coincide with Nicol et al. (18) and Astaneh et al. (19), who showed that high broiler stocking density demonstrated metabolic changes in blood biochemical markers, such as a rise in heterophils and a higher ratio of heterophils to lymphocytes, or a drop in lymphocytes, and a high heterophils/lymphocytes (H/L) ratio, which is seen as an indication of continuous stress associated with immune system performance and laying hen welfare. In addition, RBCs may be increased due to hypoxia, which resulted from high stocking density, as found previously that the splenosomatic index decreased after hypoxia and stress and led to a notable splenic constriction to enhance oxygen-carrying capacity in hypoxic conditions, which may be linked to hematopoiesis (76). The rapid increase in RBCs supported this theory even more. This finding agrees with Wells and Baldwin (77) who demonstrated this in newborn silver trevally (*Pseudocaranx dentex*) and Tambaqui (*Colossoma macropomum*), where there are fast increases in red blood cell count accompanied by a drop in splenosomatic index with high stocking density in hypoxia.

In living animals, pro-oxidant synthesis and antioxidant defenses are balanced under normal physiological circumstances. Any aberrant state elevates reactive oxygen species (ROS) and oxidative stress, which oxidize and destroy proteins and lipids inside the cell and its compartments (78). MDA, TAC, and cortisol are good indicators of oxidative stress, as their levels increased in the H group compared to the L and M groups. The results of the current experiment confirm that a high stocking density in broilers causes oxidative stress. This is caused by crowding, increased bird fights, and metabolic disruptions. These factors result in higher lipid peroxidation levels, higher oxidative damage, and reactive oxygen species (ROS) generation (16, 54). As a response to stressful conditions, the production of the antioxidant enzyme decreases, which further increases MDA production (79). In addition, our findings indicate that high stocking density led to a marked decrease in IgG in the HSD group compared to the MSD and LSD groups. However, thyroxine hormone (T4) increased in the H group compared to the L group. Verifying the results of the current experiment, in Japanese quails, the immunological response to high stocking density was significantly reduced (80).

At high stocking density, complement 3 and IgG levels decreased in terms of immunity, which is consistent with the findings of Palizdar et al. (59) who claim that broiler immunity is suppressed by high stocking density. Additionally, releasing adrenal corticosteroid hormones and somatostatin somewhat lowers immunoglobulin synthesis (81). A similar trend has been spotted previously in study reporting that stressors raised blood corticosterone levels. Corticosterone suppresses B cell synthesis of antibodies, which may account for the unfavorable association between corticosterone levels and IgG titres in HSD (82). Conversely, Li et al. (22) indicated that a higher stocking density was associated with a rise in IgG and IgM (22).

Regarding gene expression profiles, growth (IGF1 and GH), intestinal health (Muc2, Cath-B, *Calbindin*, and *Gastrotropin*), and anti-inflammatory (IL-10) genes were significantly upregulated in birds experiencing LSD than those of MSD and HSD. Meanwhile, inflammatory (IL-6, IL-8, and IL-13) markers were significantly downregulated. To our knowledge, the relationship between stock density and gene expression is not thoroughly comprehended. Profile of growth, intestinal health, and inflammatory markers (83) elaborated that the HSD group lowered the mRNA expression levels of insulin-like growth factor 1 (IGF-1). Growth factor systems, including IGF-1, are essential to the process of growth. Several studies have shown that IGF-1 regulates the growth of skeletal muscle and is involved in several signaling pathways (84). IGF-1 can both prevent muscular atrophy and stimulate hypertrophy in skeletal muscle (85). Growth hormone (GH) is the key regulator of growth rate and body composition since it affects the differentiation of muscle cells, adipocytes, and other cells that are required for development and growth (86). GH links to the GHR to promote cell division, growth, and metabolism in chickens (87), initiating hepatic function and emitting insulin-like growth factor-I (IGF-I) (88) into the bloodstream into circulation. Thus, in chickens, this promotes cell division, development, and metabolism (89). Downregulation of both genes (IGF-I and GH) in the HSD group explained the compromising growth performance based on the evidence mentioned before by Scanes (89) who documented that the key hormones that influence the expected growth of chickens are growth hormone (GH) and insulin-like growth factor-1 (IGF-1). It is widely known that HSD causes various physiological issues in chickens, such as increased oxidative stress, endocrine disruptions, immunological dysfunctions, and respiratory alkalosis (90). Stress may negatively affect immunocytes (91) and react by continually generating cytokines. IL-10 inhibits inflammation (92). When proinflammatory cytokines are produced in excess, numerous organs experience the onset of immune-mediated inflammation and subsequent fibrosis (93). On the other hand, boosting IL-10 production while receiving HSD therapy reduces inflammation and preserves immunotolerance (94). The aforementioned reasons could decipher the significant down-regulation of the IL-10 gene in HSD. However, inflammatory (IL-6, IL-8, and IL-13) markers were significantly upregulated. Regarding the expression pattern of markers, it was found to be associated with intestinal health. The HSD birds elicited significant Muc2, Cath-B, *Calbindin*, and *Gastrotropin* gene downregulation. The marked downregulation may be attributed to boosting blood flow to peripheral tissues at the expense of intestinal tissues. HSD reduces the delivery of nutrients and oxygen, compromising intestinal health and functioning (95). Additionally, overcrowding elicits limited growth space and limited feed access, which may cause nutritional deficits. Therefore, achieving a substantial decrease in the expression of the Muc2, Cath-B, *Calbindin*, and *Gastrotropin* genes is possible. In all, these metabolic and molecular alterations with poor FI may explain the considerable declines in the growth performance characteristics of birds stocked at high densities (96).

Feed costs per bird with increasing stocking density can be linked to the increased challenge of accessing feeders as stocking density rises, thereby impacting the overall cost of feeding each bird (60). Conversely, the lower stocking density group incurred decreased feed costs compared to those with higher densities, likely due to fewer birds per group. Further evidence emphasized that keeping broilers in high densities has been found to have adverse effects on the birds’ feed intake, leading to increased overall feed costs per group despite lower costs per bird (97). Similarly, the notable disparity in Total Variable Costs (TVC) and Total Costs (TC) might be attributed to varying feeding expenses per bird, as feed costs account for approximately 70% of the total production costs (98). As stocking density increased, the total fixed cost (TFC) per individual bird decreased due to the distribution of fixed costs over more birds. However, the overall TFC for the entire group increased because of the need for more resources to support the larger number of birds. Previous findings aligned with these results, indicating that the TFC, TVC and TC per group increased with higher stocking density (99).

The notable variation in body weight sales and Total Revenue (TR) can be attributed to varied Body Weight Gain (BWG) and Feed Conversion Ratio (FCR) throughout the study, as well as differences in the number of birds in each group, resulting in varying meat production per group. Consequently, with higher stocking density, there was a decrease in body weight sales and TR per bird within the group due to decreased BWG and increased FCR, while there was an increase in overall body sales per group because of greater meat produced per group. That is supported by the previous results (100). These findings are also consistent with Mitrovic (101), who observed that groups raised at a higher stocking density of 16 birds/m^2^ yielded the highest meat amount of 33 kg/group and achieved the greatest total body weight sales and TR per group.

The Net Profit (NP) values for both group and individual bird measurements for lower stocking densities can be interpreted by the formula NP = TR - TC, in which TC is higher relative to TR for groups with greater stocking densities, leading to a reduction in NP. Conversely, the group with the lowest stocking density had TR exceeding its TC, resulting in a higher NP. These findings were consistent with the results of Mitrovic (101) and Wahed et al. (99), who observed a decrease in NP per bird and group as stocking density increased.

Our results regarding productive function suggest a negative effect of stocking density on production, as indicated previously (97). This observation is consistent with Zuowei et al. (102) who reported a decline in productive performance when chickens were maintained at high stocking densities until 42 days of age. Furthermore, previous study demonstrated that the regression model for production revealed a substantial adverse impact of stocking density on final marketing body weight, accounting for 55% of the variation (103). Similarly, the results concerning economic function validate that higher marketing body weight sales positively impact NP. Furthermore, these results aligned with the unfavorable associations highlighted previously (104) regarding the influence of feed costs and previously (103) regarding the impact of stocking density on net profit (NP) within a regression model.

To sum up, the practice of keeping broilers in high stocking densities has important management implications for the broiler meat industry due to higher profits we obtained either from lowering fixed production costs (like labor, fuel, housing, and equipment) or improving meat yield (as the number of birds per unit space increases). However, poultry health has been negatively impacted by endocrinological and behavioral changes causing a negative impact on poultry health.

Thus, current technological advancements and mathematical modeling create new opportunities for automatic real-time monitoring of animal health and welfare. There are several forms of that technology, such as sensors for supervising farm environmental conditions, movement, or physiological parameters; imaging technologies like optical flow to identify issues with gait and feather pecking; besides infrared technologies to assess the thermoregulatory features and metabolic changes of birds that may be a sign of health, welfare, or management issues. The YOLOv8 object detection model is considered the most recent technological advancement to be applied to poultry welfare, particularly for broiler chickens and laying hens. It is used in artificial intelligence-driven automated poultry flock management systems to address issues like occlusions, lighting variability, and high-density flock conditions. This contributes to a scalable and dependable system for automated monitoring, decision-making, and improving poultry management efficiency.

All of these technologies have the potential to be used commercially to enhance flock management and the welfare of birds, along with increasing density efficiency and yielding more revenues without compromising performance.

The insights gained in this study shed light on the underlying gut-related regulatory mechanisms under stress conditions involved in broiler growth rates. It also emphasized the significance of gene markers (growth, intestinal health, inflammatory, and anti-inflammatory genes) to elucidate this mechanism and, as a result, establish the threshold at which growth performance is unaffected.

Sex uniformity is considered a limitation of this study. In future studies, it would be interesting to manage environmental stress in chicken farms by applying anti-stress feed additives and enriching cages using nanotechnology; this assists in increasing bird behavioral activity and welfare, besides getting use of HSD along with sex uniformity.

## 5 Conclusion

Based on this research’s findings, increased stocking density adversely affects broiler farms’ profitability due to its negative impact on broiler performance, carcass characteristics, welfare, and hematological features. In terms of gene expression patterns, birds raised at HSD had considerable downregulation of growth, intestinal health, and anti-inflammatory genes, coincided with upregulation of inflammatory markers compared to birds raised at LSD and MSD. Consequently, our study concluded that rearing in LSD up to MSD could be applied for the Avian 48 breed without compromising broiler performance.

## Data Availability

The original contributions presented in the study are included in the article/supplementary material, further inquiries can be directed to the corresponding authors.
